# All-optical phase control in nanophotonic silicon waveguides with epsilon-near-zero nanoheaters

**DOI:** 10.1038/s41598-021-88865-6

**Published:** 2021-05-04

**Authors:** Jorge Parra, Wolfram H. P. Pernice, Pablo Sanchis

**Affiliations:** 1 Nanophotonics Technology Center, Universitat Politècnica de València, Camino de Vera s/n, 46022 Valencia, Spain; 2 Institute of Physics, CeNTech, University of Münster, Heisenbergstr. 11, 48161 Münster, Germany

**Keywords:** Integrated optics, Silicon photonics

## Abstract

A wide variety of nanophotonic applications require controlling the optical phase without changing optical absorption, which in silicon (Si) photonics has been mostly pursued electrically. Here, we investigate the unique light–matter interaction exhibited by epsilon-near-zero (ENZ) materials for all-optical phase control in nanophotonic silicon waveguides. Thermo-optic all-optical phase tuning is achieved using an ENZ material as a compact, low-loss, and efficient optical heat source. For a 10-$\upmu$m-long ENZ/Si waveguide, insertion loss below 0.5 dB for the transverse electric (TE) polarization is predicted together with a high control efficiency of $\sim 0.107\uppi$ $\hbox {mW}^{-1}$. Our proposal provides a new approach to achieve all-optical, on-chip, and low-loss phase tuning in silicon photonic circuits.

## Introduction

The ability to manipulate the phase of light independently of the amplitude is a key challenge in the development of silicon (Si) photonic integrated circuits (PICs). Pure-phase control enables a broad realm of nanophotonic applications such as programmable photonic circuits^1^, nanophotonic phased arrays^2^, deep learning^3^, and quantum computing^4^, to name a few. Most widespread methods for electrically inducing an optical phase shift with negligible or low optical loss rely either on the silicon thermo-optic coefficient by means of microheaters^5^ or on p-i-n injection type devices exploiting the silicon plasma dispersion^6^. Optical phase modulation by means of Pockels effect has also been investigated in silicon waveguides by breaking the crystal symmetry with a highly stressed layer deposited on top^7,8^. Alternatively, quadratic electro-optic or DC Kerr effect has been demonstrated using a p-i-n structure under reverse bias^9,10^. However, to date, the phase modulation efficiency is rather low with both approaches. Therefore, intense research is devoted to integrating silicon photonic devices with complementary-metal-oxide-semiconductors (CMOS) materials, such as materials with large Pockels coefficient^11^ or 2D materials^12^, with the aim of achieving faster and more efficient phase tuning driven by an electrical signal.

Pure-phase all-optical modulation can also be obtained by exploiting non-linear effects. Third-order nonlinearities are dominant in silicon due to its crystalline nature. Therefore, all-optical phase control may be achieved by means of the Kerr effect. However, the non-linearity is weak and narrowband cavities or high-power optical signals are usually required to enhance light-matter interaction (LMI)^13–19^. Furthermore, such an approach is not free of optical loss due to the associated large two-photon absorption (TPA) and single-photon absorption (SPA) processes as well as the generation of free carries^20^. Thereby, the integration of CMOS-compatible materials in silicon photonics also stands out as a promising route towards all-optical phase modulation. In this context, epsilon-near-zero (ENZ) photonics holds promise for overcoming some of the limitations found in photonic integrated devices platforms^21^.

Materials featuring near-zero permittivity at telecom wavelengths such as transparent conducting oxides (TCOs) have pushed silicon photonic devices like modulators to unprecedented and record-breaking performances^21^. The optical properties of TCOs can be largely tuned by changing their carrier concentration. By embedding the ENZ material in a metal-oxide-semiconductor (MOS) structure, micron-scale electro-absorption ENZ/Si modulators have been demonstrated exhibiting GHz-fast modulation rates, low-driving voltages ($\sim 2$ V), and optical broadband operation due to the non-resonant operation^22,23^. Such values could be further improved, achieving high-speed over 40 GHz together with energy consumption as low as 0.4 fJ/bit by using a high-mobility TCO as ENZ material^23^. Recently, Mach–Zehnder interferometric modulators using a sub-$\lambda$ ENZ/Si electro-static phase shifter have exhibited an ultra-low $\hbox {V}_{\uppi }$L of 95 V$\upmu$m with prospectsto reduce even further this value down to 3 V$\upmu$m^24^. Moreover, ultra-compact photonic memories^25^ and non-volatile switches^26^ could be achieved by using the ENZ material in a flash-like structure similar to electronic memories. On the other hand, very recently, an all-optical absorption ENZ/Si switch exploiting the large optical nonlinearity of TCOs in its ENZ regime has been proposed. Modulation strengths of 15.9 dB/$\upmu$m over a large bandwidth and ultra-fast switching of 230 fs together with ultra-low energies of 13.5 fJ have been predicted^27^.

In the present work, we investigate the utilization of an ENZ material to enable all-optical phase control in nanophotonic silicon waveguides. A phase shift for TE polarization is induced by exploiting the silicon thermo-optic coefficient. Such phase shift is optically controlled by means of a cross-polarized TM signal. The heat originates from a thin layer of ENZ material deposited on top of the waveguide acting as an optical heat source. The pass polarizer behavior of the hybrid ENZ/Si waveguide provides low optical loss for TE phase shifted signal but efficient optical-heat conversion for the TM one.

**Figure 1:**
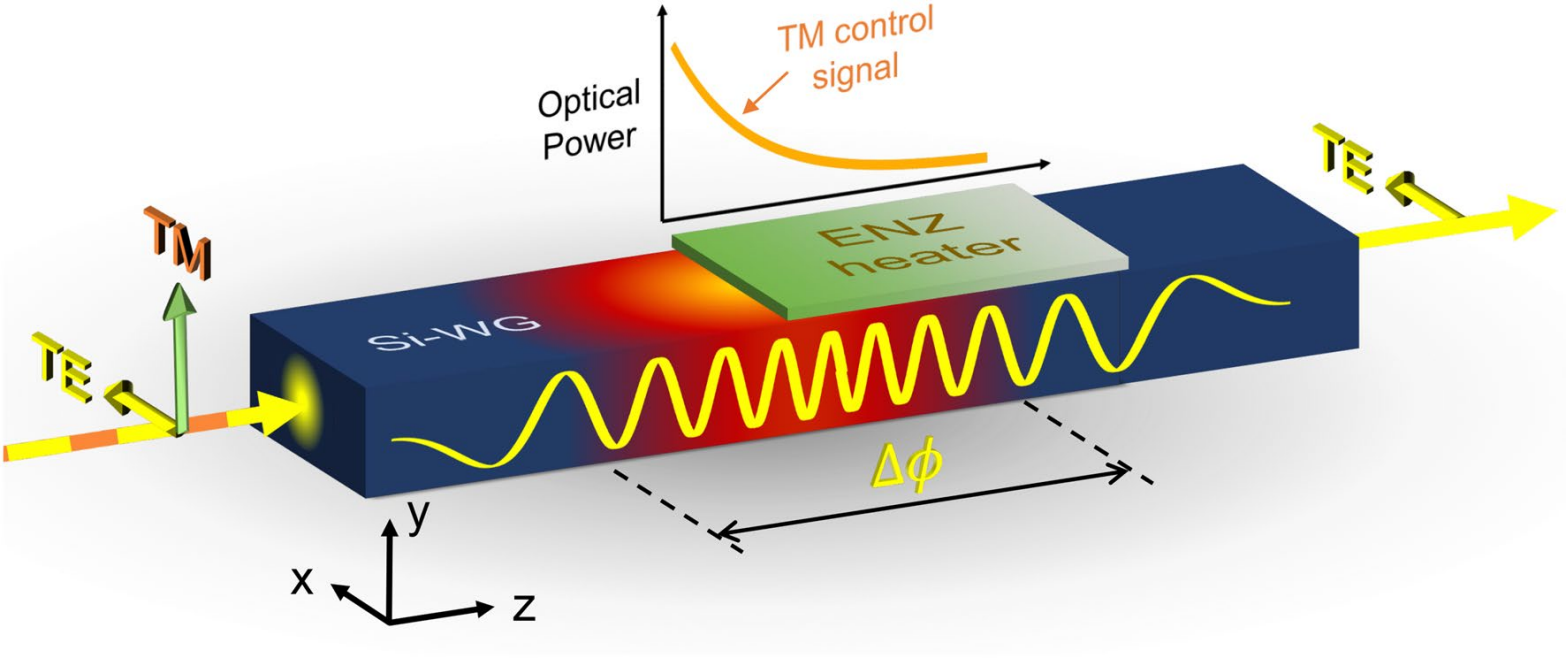
Illustration of the proposed all-optical phase shifter. The optical heater is an ENZ material that heats up due to the absorbed optical power of the TM polarized mode. The phase shift is induced for TE polarization by exploiting the silicon thermo-optic coefficient and leads to low optical loss due to the pass polarizer operation of the hybrid waveguide section.

## Results

### Working principle of thermo-optic tuning in silicon waveguides with optical ENZ nanoheaters

Figure 1 shows a schematic of the proposed all-optical phase shifter. Absorptive materials can work as optical heat sources according to the radiative transport equation (RTE) and the resulting energy conservation law^28,29^. In that case, the heat source *Q* is driven by the energy of the incident beam, i.e., $Q \propto \kappa I$, where $\kappa$ is the absorption coefficient of the lossy medium and *I* the optical intensity impinging onto the medium. Particularly, in a waveguide, $\kappa$ and *I* correspond to the effective extinction coefficient, $\kappa _{\text {eff}}$, and the intensity of the mode propagating through the waveguide, respectively. The value of $\kappa _{\text {eff}}$ is the result of the LMI between the optical mode and the lossy heater. Hence, the key challenge to achieve low optical loss for the phase shifted mode in combination with a highly efficient optic-heat conversion would be to link the optical loss mainly to polarization and not to the intrinsic absorption of the heater. A polarization dependent loss operation could be achieved by reducing the intrinsic loss of the heater and obtaining a strong LMI for TM polarization but weak for TE polarization. Such behavior can be reached by using a heater based on an ENZ material ($\varepsilon '=0$) with relatively low optical loss, $\varepsilon ''$. In that case, the difference in terms of LMI between both polarizations will be determined by the boundary conditions. For TM polarization, the major component of the electric field, $E_{y}$, is oriented normally to the interface between the ENZ heater and the silicon waveguide. Thus, giving the continuity of the displacement, the electric field at the interface of the ENZ material is $E_{ENZ}=\left|\varepsilon _{Si}\right|/\left|\varepsilon _{ENZ}\right|E_{Si}$. Note that, for $\varepsilon _{ENZ}'=0$ and very low $\varepsilon ''$ ($\varepsilon ''<1$), the value of $E_{ENZ}$ is greatly enhanced, thus giving rise to strong LMI. On the other hand, for TE polarization most of the electric field, $E_{x}$, is tangential to the ENZ/Si interface. Then, the boundary condition for this case is $E_{ENZ}=E_{Si}$. Therefore, there is no electric-field enhancement and thus, since most of the optical mode is confined within the silicon waveguide, there is very weak LMI. Based on these considerations we design an optimal ENZ layer for such purposes. The considered ENZ/Si waveguide comprises a standard 500 nm $\times$ 220 nm nanophotonic waveguide made from silicon-on-insulator (SOI) with a thin ENZ layer on top. We assume that the waveguide is covered with a 1-$\upmu$m-thick $\hbox {SiO}_{{2}}$ upper-cladding as in typical silicon PICs to provide protection against the environment. Information about the material parameters can be found in the “Methods” section.

### Optimal ENZ layer: thickness and loss

**Figure 2:**
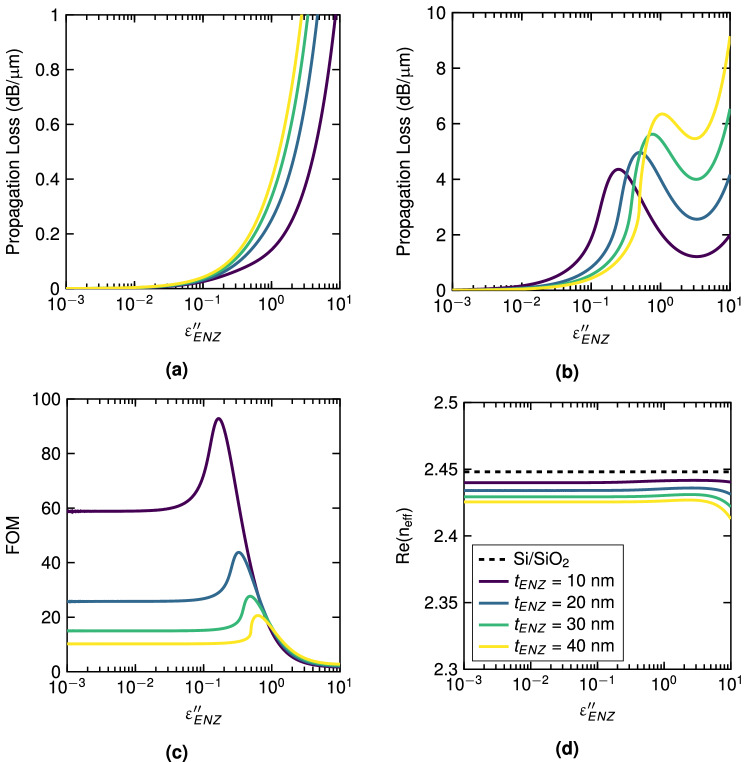
Derived parameters from the complex effective index of TE and TM modes of the hybrid ENZ/Si waveguide as a function of the loss of the ENZ material, $\varepsilon _{ENZ}''$, and for different ENZ layer thickness, $t_{ENZ}$. Propagation loss for (**a**) TE and (**b**) TM polarized modes. (**c**) Figure of merit. (**d**) Real part of the effective index for the TE mode. Values are given for $\lambda =1550$ nm while imposing $\varepsilon _{ENZ}'=0$.

The optimal thickness and loss of the ENZ layer are obtained by assessing the figure of merit $FOM = \kappa _{\text {eff}}^{TM}/\kappa _{\text {eff}}^{TE}$. To this end, we obtained the complex effective index for the TE and TM mode of the hybrid waveguide by 2D finite element (FEM) simulations (see “Methods” section for details). Figure 2 shows the values for different $\varepsilon _{ENZ}''$ and layer thicknesses. For the analysis, the considered fundamental TM mode corresponds to the solution with highest effective index when the ENZ/Si waveguide supports multiple propagation TM-polarized modes. For TE polarization, the propagation loss increases with the thickness and the intrinsic loss of the ENZ material is comparable to lossy dielectrics (Fig. 2a). In contrast, for TM polarization, we observe a maximum for the different thicknesses (Fig. 2b). This maximum is found in the region of $\varepsilon _{ENZ}''$ values in which the loss is the result of strong coupling between $\varepsilon _{ENZ}''$ and LMI since LMI is related to $1/|\varepsilon _{ENZ}|$. For $\varepsilon _{ENZ}''$ values beyond the maximum, the LMI reduces more than $\varepsilon _{ENZ}''$ is increased, resulting in an overall reduction of the optical loss. On the other hand, for values of $\varepsilon _{ENZ}''>3$ approx., the intrinsic loss of the ENZ material becomes the dominant factor on the optical loss. As shown in Fig. 2c, the best FOM ($\sim 94$) is achieved for $\varepsilon _{ENZ}''=0.17$ and 10-nm-thick layer, which corresponds to a propagation loss of 0.041 dB/$\upmu$m and 3.84 dB/$\upmu$m for TE and TM modes, respectively. Regarding the impact of the ENZ layer on the optical phase, the real part of the effective index for the TE mode is shown in Fig. 2d. The effective index of a silicon waveguide without ENZ material is kept as a reference value. We note that the impact of the ENZ material is very small in a wide range of $\varepsilon _{ENZ}''$ values and for the different thicknesses due to the weak LMI.

### Coupling loss and higher-order modes

**Figure 3:**
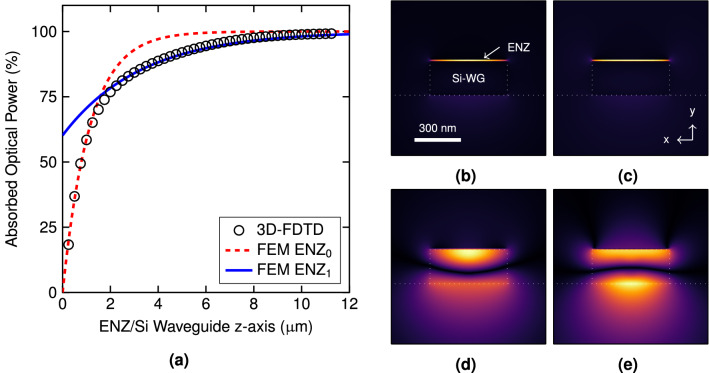
(**a**) Absorbed optical power by the ENZ layer along the ENZ/Si waveguide obtained by 3D-FDTD and fitting with the values of ENZ modes given by FEM. Normalized (**b**) $|E_{y}|$ and (d) $|E_{z}|$ field components of the $\hbox {ENZ}_{{0}}$ mode. (**c**,**e**) Same as (**b**,**d**) but for the $\hbox {ENZ}_{{1}}$ mode. The complex effective index (propagation loss) is $1.61+j0.109$ (3.84 dB/$\upmu$m) and $1.499+j0.038$ (1.32 dB/$\upmu$m) for the $\hbox {ENZ}_{{0}}$ and $\hbox {ENZ}_{{1}}$ modes, respectively. Simulations were carried out at $\lambda =1550$ nm and for a 10-nm-thick ENZ layer with $\varepsilon _{ENZ}=j0.17$.

Low loss coupling between the photonic and ENZ/Si waveguide mode is also of high importance to efficiently transform the optical power of the TM polarized signal into heat. We investigate the coupling loss between the silicon and the optimal ENZ/Si waveguide using 3D finite-difference time-domain (3D-FDTD) simulations (see “Methods” section for details). For TM polarization, a high coupling efficiency of $\sim 90\%$ (0.45 dB) was obtained (see Supplementary Movie). However, the absorbed power along the propagation direction (Fig. 3a) revealed the excitation of two TM modes, hereinafter called ENZ modes because the light is tightly confined in the ENZ layer. For the first mode ($\hbox {ENZ}_{{0}}$), the optical absorption is in good agreement with the propagation loss given in Fig. 2b until $z\approx 1.5$ $\upmu$m. For larger values, the attenuation fits the second ENZ mode ($\hbox {ENZ}_{{1}}$) that features lower propagation loss (1.32 dB/$\upmu$m). Their complex effective indices are $1.61 + j0.109$ and $1.499+j0.038$, respectively. The $E_{y}$ field component of the $\hbox {ENZ}_{{0}}$ mode (Fig. 3b) is very similar to the $\hbox {ENZ}_{{1}}$ (Fig. 3c) and we attribute this fact the reason to excite both modes in the hybrid waveguide. Despite the large similarity of the $E_{y}$, these modes are different as it can be noted by inspecting the $E_{z}$ component (Fig. 3d,e). On the other hand, the nature of the ENZ modes is attributed to be similar to the so-called short-range surface plasmon-polariton (SR-SPP) waves, which can be found in very thin metallic layers ($\varepsilon '<0$) surrounded by dielectric materials ($\varepsilon '>0$)^30^.

Nevertheless, the impact of the higher-order ENZ mode is small. In order to absorb $\sim 99\%$ of the optical power, only 10 $\upmu$m of length are required for the ENZ/Si waveguide. Moreover, the coupling loss for TE polarization was also verified to be negligible by using 3D-FDTD simulations and insertion loss was in good agreement with the propagation loss calculated by FEM. For such length, the resulting overall loss for the TE mode is $\sim 0.4$ dB.

**Figure 4:**
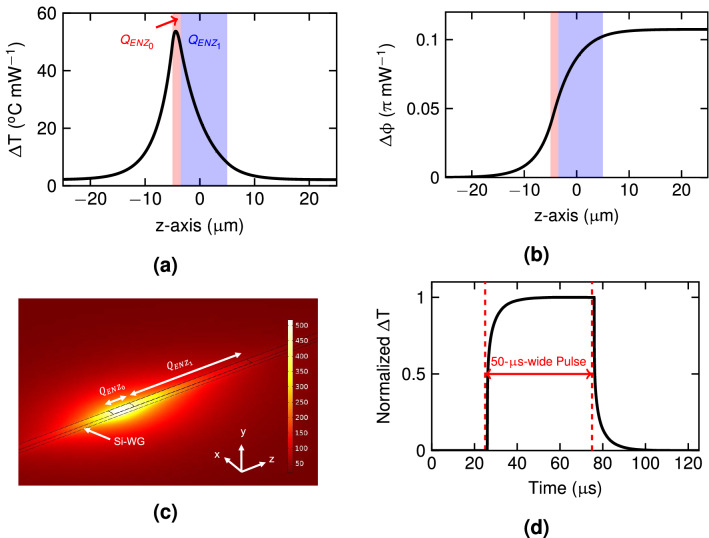
(**a**) Normalized temperature increment in the silicon waveguide, $\Delta T$, and (**b**) phase increment of the TE mode, $\Delta \phi$, along the z-axis (propagation direction). (**c**) Temperature distribution, in $^\circ$C, for 9.5 mW in the TM polarized signal. The $\hbox {SiO}_{{2}}$ upper-cladding is hidden. (**d**) Time-domain response of the silicon waveguide peak temperature upon a 50-$\upmu$s-wide square pulse.

### Thermo-optic response

The thermal response of such ENZ/Si phase shifter was obtained by modeling the ENZ as a heat source and solving the heat conduction equation (see “Methods” section for details). Figure 4a shows the normalized temperature increment in the silicon waveguide along the z-axis (light propagation direction). The accumulated phase shift of the TE mode along the waveguide is shown in Fig. 4b (see “Methods” section for details). Since there is a thermal gradient along the silicon waveguide, the phase shift begins to occur some microns before the ENZ/Si waveguide. On the other hand, the relation between the induced phase shift over the TE mode and the power of the TM polarized signal is $\sim 0.107$ $\uppi$ mW${^{-1}}$, resulting in an optical power for a $\uppi$ phase shift of around 9.5 mW. The temperature distribution for the whole structure for such optical power is depicted in Fig. 4c. Most of the heat is concentrated in the first microns around the ENZ/Si waveguide due to the high propagation loss of the $\hbox {ENZ}_{{0}}$ mode that leads to the absorption of most of the power. Moreover, this behavior is enhanced due to the high thermal conductivity of silicon, which generates a strong thermal gradient around the peak temperature. The temporal switching response was obtained from the evolution of the peak temperature within the silicon waveguide in the time-domain upon a square pulse (Fig. 4d). The rise/fall time is around 5.3 $\upmu$s using the 10-90% rule, in good agreement with heater-based thermo-optic phase shifters and limited by the low thermal diffusivity of the surrounding $\hbox {SiO}_{{2}}$.

## Discussion

For practical implementation, an ENZ heater exhibiting such optical properties at telecom wavelengths could be implemented utilizing TCOs or by forming a mixing media based on the effective medium theory formed by a host dielectric with metal nanoparticles^21^. However, TCOs provide a more practical and versatile way to obtain such ENZ heaters due to the different CMOS-compatible deposition techniques and the ability to precisely deposit thin films and tailor the optical properties during the deposition process or with a post-annealing treatment^31–33^. The permittivity of TCOs at telecom wavelengths is described by the Drude model^31^

1 $$\begin{aligned} \varepsilon =\varepsilon _{\infty }\left( 1-\dfrac{\omega _{p}^{2}}{\omega ^{2}+j\omega \Gamma }\right) . \end{aligned}$$

To meet the optimal condition $\varepsilon _{ENZ}=j0.17$, the TCO should exhibit $\omega _{p}\approx 1.126\times 10^{15}$ rad/s and $\Gamma \approx 0.045\omega _{p}$, considering a value of 3.8 for the $\varepsilon _{\infty }$^31^. In Ref.^31^, the authors report $\omega _{p}=1.2\times 10^{15}$ rad/s and $\Gamma = 0.13\omega _{p}$ for indium tin oxide (ITO). Hence, it seems reasonable to accomplish such optimal material taking into account the rapid advances in the synthesis of TCOs^32,33^. Moreover, ITO has been already experimentally demonstrated as a microheater with good thermal stability for temperatures up to $\sim 650$ $^\circ$C^34,35^.

The underlying heat mechanism is the same as in thermo-optic phase shifters based on Joule heating. Therefore, similar approaches can be followed to improve the power consumption or time response. The maximum temperature could be lowered by obtaining a longer but more uniform temperature increment according to Eq. (5). To this end, the value of $\kappa _{\text {eff}}$ could be reduced [see Eqs. (3), (4)] by using an ENZ material with lower $\varepsilon ''$ or introducing a gap between the silicon waveguide and the heater. However, this would be detrimental by increasing the power consumption when using the same surrounding materials. A drastic reduction of the optical power could be obtained by creating air-trenches around the ENZ/Si waveguide^36^. However, it should be noted that the time response would also be slowed down by a similar margin. On the other hand, one way to improve the time response would be to use a heat sink or engineering the shape of the excitation pulse^5^. Yet, both approaches will increase the peak optical power, which might give rise to silicon nonlinear effects. Therefore, the optimization process will depend on the target application.

In conclusion, we have shown the potential of utilizing ENZ materials for optical phase control in silicon waveguides with ultra-compact lengths and almost negligible insertion losses. Our approach exploits the large change in terms of LMI between TE and TM polarized optical modes when using a thin ENZ layer ($\sim 10$ nm) with relatively low optical loss ($\varepsilon _{ENZ}''\approx 0.17$). Under these conditions, the ENZ layer is almost transparent for TE but highly lossy for TM. Therefore, the ENZ layer acts as an efficient optical heat source and drives a thermo-optic phase shift of a TE polarized signal with an ENZ/Si waveguide of only 10 $\upmu$m length and with an efficiency of $\sim 0.107$ $\uppi$ mW${^{-1}}$. Our proposed device underlines the prospects of ENZ/Si integrated photonics and provides a new approach towards all-optical phase-based applications for the silicon photonic platform.

## Methods

### Optical and thermal constants used for simulations

The refractive index and thermal constants used for optical and thermal simulations, respectively, are shown in Table 1. The refractive index is shown at 1550 nm. For the thermal constants, the ENZ material was considered to be ITO. For the silicon thermo-optic coefficient we used $1.86\times 10^{-4}$ $\hbox {K}^{-1}$^37^.

**Table 1:** Optical and thermal constants used for simulations.

Material	Refractive index	Thermal conductivity (W $\hbox {m}^{-1}$ $\hbox {K}^{-1}$)	Specific heat capacity (J $\hbox {kg}^{-1}$ $\hbox {K}^{-1}$)	Density (kg $\hbox {m}^{-3}$)
Si	3.476	148	703	2230
$\hbox {SiO}_{{2}}$	1.444	1.38	709	2203
ENZ	$\sqrt{j\varepsilon _{ENZ}''}$	11	1290	7100

### 2D finite element method simulations

**Figure 5:**
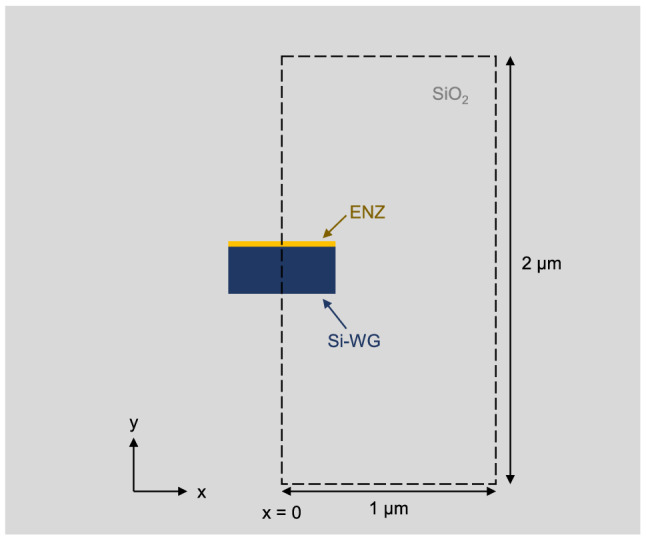
Illustration of the simulated region by 2D-FEM to obtain the optical modes of the ENZ/Si waveguide. The ENZ layer was removed in the case of the Si-only waveguide.

The optical modes of the hybrid ENZ/Si and Si-only waveguide were obtained by 2D-FEM simulations with RSoft FemSIM simulation tool. Figure 5 shows the domain used for such simulations. This comprised a region from $x = 0$ to $+\,1$ $\upmu$m and $y = -\,1$ to $+\,1$ $\upmu$m. Symmetric boundary conditions were applied at $x = 0$ $\upmu$m to reduce both simulation time and memory considering the symmetry in the *x*-axis of the waveguide. The grid size for the *x*-axis was set to 20 nm. Conversely, a non-unfirm mesh was used for the *y*-axis to obtain a good accuracy within the ENZ layer. In this case, the grid size was set to 5 nm with a minimum subdivision of 10 points.

### 3D finite-difference time-domain simulations

**Figure 6:**
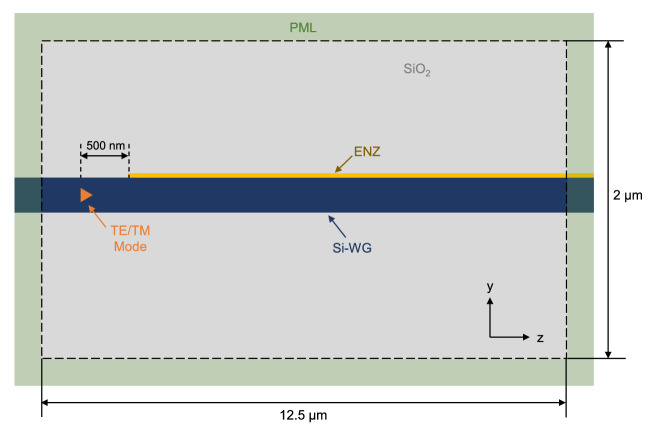
Illustration (not to scale) of the simulated region (YZ plane) by 3D-FDTD. Monitors are not represented for better clarity.

3D-FDTD simulations with RSoft FullWAVE simulation tool were carried out to investigate the coupling efficiency and optical loss of the ENZ/Si waveguide. The structure comprised an initial section of a 500-nm-long Si-only waveguide followed by an 11.5-$\upmu$m-long ENZ/Si waveguide using a 10-nm-thick ENZ layer with $\varepsilon _{ENZ}=j0.17$ ($n_{ENZ}=0.2915+j0.2915$). Both waveguide sections were surrounded by $\hbox {SiO}_{{2}}$. Figure 6 shows the simulated domain in the YZ plane. The XZ plane is similar as shown in Fig. 5. The simulation region comprised in the *x*-axis from $x = 0$ to $x = +\,1$ $\upmu$m, in the $y = -\,1$ to $y = +\,1$ $\upmu$m, and in the $z = -\,0.5$ to $z = +\,12$ $\upmu$m. Perfectly matched layer (PML) boundary conditions were applied to all the boundaries with the aim of avoiding reflections, except at $x = 0$ in which symmetric boundary condition was applied. Each PML boundary was formed by 10 PML cells. The waveguide was extended completely through the PML region to avoid any reflection. For the *x*- and *z*-axis a grid size of 20 nm was set. Similar to FEM simulations, a non-uniform mesh was applied in the ENZ layer with a grid size of 5 nm and a minimum division of 10 points, i.e., 1 nm of resolution. The TE/TM polarized mode of the Si-only waveguide obtained by 2D-FEM was used as optical excitation. The launch field was located at $z = 0$ to avoid any interaction with the PML of the $-z$ boundary. Finally, several monitors were placed along the ENZ/Si waveguide to measure the optical power. The monitors’ size was 1 $\upmu$m $\times$ 1 $\upmu$m and the distance between two consecutive monitors was 250 nm.

### Definition of the ENZ heat source and thermal simulations

The optical ENZ heat source, $Q_{ENZ}$, can be described as:

2 $$\begin{aligned} Q_{ENZ} = \dfrac{P_{TM}\Gamma }{w_{ENZ}t_{ENZ}}\dfrac{\partial }{\partial z}\left[ 1 - P_{WG}(z) \right] \end{aligned},$$

where $P_{TM}$ is the optical power of the photonic TM mode, $\Gamma$ is the coupling efficiency between the photonic and ENZ/Si waveguide, $w_{ENZ}$ and $t_{ENZ}$ are the width and thickness of the ENZ layer, respectively, and $P_{WG} (z)$ is the normalized optical power within the ENZ/Si waveguide along the propagation direction. Considering that $P_{WG} (z)$ fits the excitation of the ENZ modes in the short and long region, respectively, then $Q_{ENZ}=Q_{ENZ_{0}}+Q_{ENZ_{1}}$. The value of $Q_{ENZ_{0/1}}$ is:

3 $$\begin{aligned} Q_{ENZ_{0}} = \dfrac{P_{TM}\Gamma }{w_{ENZ}t_{ENZ}}\dfrac{4\pi \kappa _{\text {eff}}^{ENZ_{0}}}{\lambda }\exp \left[ -\dfrac{4\pi \kappa _{\text {eff}}^{ENZ_{0}}}{\lambda }(z-z_{0}) \right] \end{aligned},$$

and

4 $$\begin{aligned} Q_{ENZ_{1}} = P_{WG}(z_{1})\dfrac{4\pi \kappa _{\text {eff}}^{ENZ_{1}}}{\lambda }\exp \left[ -\dfrac{4\pi \kappa _{\text {eff}}^{ENZ_{1}}}{\lambda } \left( z-z_{1}\right) \right] . \end{aligned}$$

The ENZ/Si waveguide is assumed to start at $z = z_{0}$ and $z_{1}$ is the point from which the optical attenuation is described by the $\hbox {ENZ}_{{1}}$ mode.

**Figure 7:**
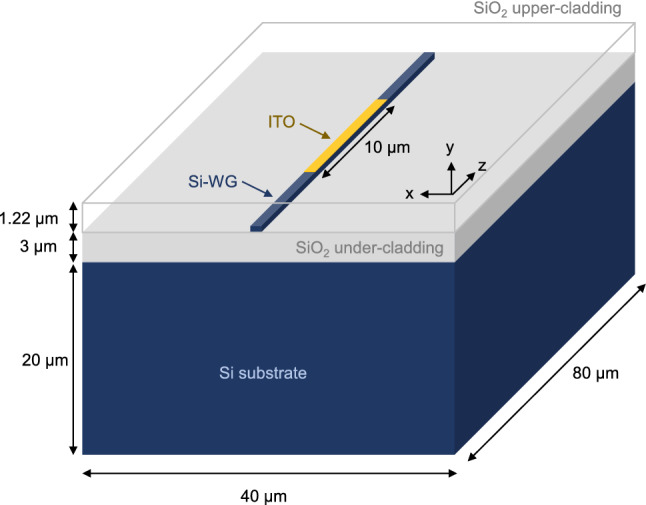
Illustration (not to scale) of the structure used for thermal simulations. The $\hbox {SiO}_{{2}}$ upper-cladding is set transparent for better clarity of the waveguide.

3D thermal simulations were conducted by solving the heat conduction equation in the steady-state and the time-domain with COMSOL Multiphysics simulation tool. Contribution from radiation heat transfer was assumed to be negligible. Figure 7 illustrates the structure used for such simulations. The ENZ/Si waveguide was defined using a 10-nm-thick ITO layer with 10 $\upmu$m of length. The hybrid waveguide was placed in the middle (*z*-axis) of the simulation region and the length of the Si-only waveguide extended to 80 $\upmu$m. The influence of the silicon substrate was taken into account setting a dimension (width $\times$ height) of 40 $\upmu$m $\times$ 20 $\upmu$m. The height of the $\hbox {SiO}_{{2}}$ under- and upper-cladding was 3 $\upmu$m and 1.22 $\upmu$m (1 $\upmu$m + Si-wg height). A tetrahedral non-uniform mesh was used comprising different element sizes. The ITO layer (heat source) was discretized with elements of sizes between 1 nm and 50 nm. The silicon waveguide utilized elements of between 50 and 200 nm. Finally, the remaining domains (substrate, under- and upper-cladding) were formed by elements of between 500 nm and 3 $\upmu$m. Convective heat flux was set as a boundary condition on top of the upper-cladding with a heat transfer coefficient $h = 5$ W $\hbox {m}^{-2}$ $\hbox {K}^{-1}$. Temperature boundary condition for the remaining boundaries was used by setting $T = 293.15$ K (20 $^\circ$C). The heat source in the steady-state was defined following Eqs. (3) and (4). For time-domain simulations, that value was used for the amplitude of a 50-$\upmu$s-wide square pulse using a time step of 25 ns.

### Phase shift calculation

The phase shift induced to the optical mode, $\Delta \phi$, due to a temperature increment of the silicon waveguide, $\Delta T_{Si}$, and over a certain length, *L*, is obtained as:

5 $$\begin{aligned} \Delta \phi = \dfrac{2\pi }{\lambda }\dfrac{\partial n_{\text {eff}}}{\partial T} \int _{0}^{L}\Delta T_{Si}(z) dl \end{aligned},$$

where $\partial n_{\text {eff}} / \partial T$ is the effective index dependence with the temperature due to the silicon thermo-optic coefficient. For a standard silicon waveguide surrounded by $\hbox {SiO}_{{2}}$ the value of $\partial n_{\text {neff}} / \partial T = 2\times 10^{-4}$ $\hbox {K}^{-1}$. The same value was obtained for the ENZ/Si waveguide. As a result, the integral of Eq. (5) should be equal to 3875 K $\upmu$m to obtain a $\uppi$ phase shift at $\lambda =1550$ nm.

## Supplementary Information

Supplementary Information.

Supplementary Movie.
