# Supplementary material for: All-optical phase control in nanophotonic silicon waveguides with epsilon-near-zero nanoheaters
Source: Sci Rep. 2021 May 4;11:9474. doi: 10.1038/s41598-021-88865-6 (PMC8096950; doi:10.1038/s41598-021-88865-6)
Supplement: Supplementary file 1 — Supplementary Information. [file 41598_2021_88865_MOESM1_ESM.docx]

**All-optical phase control in nanophotonic silicon waveguides with epsilon-near-zero nanoheaters**

**Jorge Parra**^1^**, Wolfram H. P. Pernice**^2^**, and Pablo Sanchis**^1,*^

^1^Nanophotonics Technology Center, Universitat Politècnica de València, Camino de Vera s/n, 46022 Valencia, Spain

^2^Institute of Physics, CeNTech, University of M¨unster, Heisenbergstr. 11, 48161 Münster, Germany

*pabsanki@ntc.upv.es

The supplementary material contains a video file which shows a lateral view of the 3D-FDTD simulation of the silicon waveguide coupled to the optimal ENZ/Si waveguide for TM polarization.
